# Associations of adherence to the DASH diet and the Mediterranean diet with chronic obstructive pulmonary disease among US adults

**DOI:** 10.3389/fnut.2023.1031071

**Published:** 2023-02-02

**Authors:** Jingli Wen, Shujun Gu, Xinyu Wang, Xu Qi

**Affiliations:** Department of Respiratory and Critical Medicine, First Affiliated Hospital, Nanjing Medical University, Nanjing, Jiangsu, China

**Keywords:** COPD, DASH diet, Mediterranean diet, pulmonary function, NHANES

## Abstract

**Background:**

The Dietary Approaches to Stop Hypertension (DASH) and the Mediterranean diet are associated with reduced cardiovascular, tumor, and diabetes risk, but the effect on chronic obstructive pulmonary disease (COPD) is uncertain.

**Objective:**

To investigate the association of the DASH diet and the Mediterranean diet with the risk of COPD in American adults.

**Methods:**

This cross-sectional study included 28,605 participants from the National Health and Nutrition Examination Survey (NHANES) 1999–2018 survey cycle who had complete dietary and other questionnaire data. The scores of healthy eating patterns (the DASH diet and the Mediterranean diet) were derived from a 24-h dietary recall interview [individual food and total nutrient data from NHANES and food pattern equivalents data from the United States Department of Agriculture (USDA)]. The primary outcome was the prevalence of COPD. COPD was defined based on participants self-reported whether or not a doctor or health professional had diagnosed chronic bronchitis or emphysema. Secondary outcomes were lung function and respiratory symptoms. All analyses were adjusted for demographics and standard COPD risk factors (primary tobacco exposure, secondhand smoke exposure, and asthma).

**Results:**

This study included 2,488 COPD participants and 25,607 non-COPD participants. We found that a higher DASH diet score was associated with a lower risk of COPD [odds ratio (OR): 0.83; 95% confidence interval (CI): 0.71–0.97; *P* = 0.021]. This association persisted in several subgroups [men (OR: 0.73; 95% CI: 0.58–0.93; *P* = 0.010), relatively young (OR: 0.74; 95% CI: 0.55–1.01; *P* = 0.050), and smoker (OR: 0.82; 95% CI: 0.67–0.99; *P* = 0.038)]. In contrast, the Mediterranean diet score was not significantly associated with COPD prevalence in this large cross-sectional analysis representative of the US adult population (OR: 1.03; 95% CI: 0.88–1.20; *P* = 0.697). In addition, we found a correlation between DASH diet adherence and lung function [β: −0.01; 95% CI: −0.01–0.00; *P* = 0.003 (FEV1: FVC)] or respiratory symptoms [OR: 0.80; 95% CI: 0.73–0.89; *P* < 0.001 (dyspnea); OR: 0.80; 95% CI: 0.70–0.91; *P* = 0.002 (cough); OR: 0.86; 95% CI: 0.74–0.99; *P* = 0.042 (expectoration)], especially in non-COPD populations.

**Conclusion:**

A higher DASH diet score was associated with improved COPD prevalence, lung function and respiratory symptoms. This new finding supports the importance of diet in the pathogenesis of COPD and expands the scope of the association of the DASH diet score with major chronic diseases.

## Introduction

Chronic obstructive pulmonary disease (COPD) is the leading cause of global morbidity, mortality and health care burden, affecting about 10 per cent of the adult population (1) and causing 3.23 million deaths in 2019 (2). Due to chronic exposure to risk factors (3, 4) and changes in the age structure of the world population (5), the burden of COPD is expected to increase (6). Although COPD is heterogeneous, it has common characteristics, including persistent airflow obstruction and respiratory symptoms. Many people who are diagnosed with chronic obstructive pulmonary disease in late adulthood begin to develop symptoms in middle age, which may last several years before diagnosis (7). A better understanding of the symptoms of early chronic obstructive pulmonary disease helps to increase awareness of the early life factors that contribute to the risk of chronic obstructive pulmonary disease. Therefore, early treatment before developing into a serious irreversible disease can minimize disability. In addition, chronic obstructive pulmonary disease is caused by exposure to respirable particles, such as cigarette smoke and air pollutants, coupled with genetic, developmental and social factors (8). Although quitting smoking is essential for the prevention of COPD (2), it is also important to recognize the effects of other risk factors, such as diet, on COPD (9).

Diet has been recognized as a modifiable risk factor for the development and progression of chronic diseases (10), and recent evidence is increasingly pointing to the role of diet in obstructive pulmonary disease, including COPD (9, 11). Previous dietary studies have focused on specific foods and nutrients, including antioxidants, vitamins, fatty acids, meat, dietary fiber, fruits, and vegetables. Although the results of these studies vary, it seems that eating a diet rich in antioxidants, vitamins, omega-3 fatty acids, fiber, fruits, and vegetables, as well as low-processed meat can provide some protection against chronic obstructive pulmonary disease (12–18). However, some studies have shown that individual nutrients or selected foods have no significant effect on COPD or lung function (19–21). In this case, dietary patterns provide an overview of diet and are a good way to assess the relationship between diet and COPD. Healthy dietary models described in the 2020–2025 Dietary Guidelines for Americans (22) and recommended by professional associations (23), including the Dietary Approaches to Stop Hypertension (DASH) and the Mediterranean diet, have been shown to improve cardiovascular risk factors and their prevalence (24–28), reduce the incidence of cancer and metabolic diseases (29–32) and reduce mortality (33–35), but there are relatively few studies on the relationship between these two dietary patterns and COPD.

The main purpose of this study was to use comprehensive data from the National Health and Nutrition Examination Survey (NHANES) from 1999 to 2018 to assess the relationship between the DASH diet or the Mediterranean diet and COPD prevalence. In addition, this study explored the relationship between these two dietary patterns and lung function or respiratory symptoms (dyspnea, cough, and expectoration).

## Materials and methods

### Data and population

**FIGURE 1:**
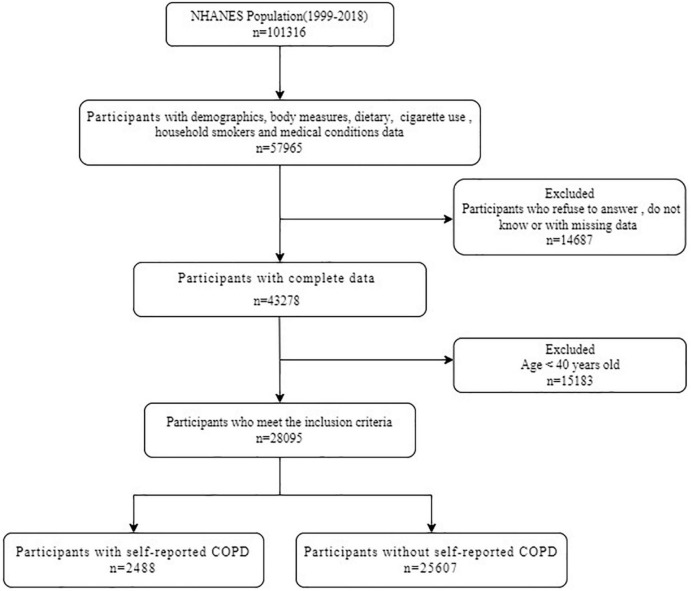
Flow diagram of study participants. NHANES, National Health and Nutrition Examination Survey; COPD, chronic obstructive pulmonary disease.

The data are extracted from NHANES, a cross-sectional survey of a nationally representative sample of the non-institutionalized civilian population in the United States. NHANES is based on a stratified multi-stage probabilistic sampling design. More information about the design and procedures of NHANES can be found on the website of the Centers for Disease Control and Prevention (CDC) (36). The researchers assessed the health and nutritional status of participants by collecting demographic data, diet data, anthropometric data, laboratory data, and questionnaire data. The NHANES survey was approved by the Research Ethnics Review Board of the National Centre for Health Statistics, and all participants in NHANES provided informed consent. Figure 1 shows the selection of study participants for analysis. A total of 28,095 participants were analyzed after excluding participants under the age of 40 and those who lacked information about nutritional intake, history of COPD and related covariates (Figure 1).

### Outcomes

#### Primary outcome

The main outcome of the study was COPD, where COPD was defined as having been diagnosed with “chronic bronchitis or emphysema” because data on the problem existed from 1999 to 2018 (37).

#### Secondary outcomes

Secondary study outcomes included lung function and respiratory symptoms (dyspnea, cough, and expectoration).

During the 2007–2012 NHANES, participants between the ages of 6 and 79 received vital capacity measurements, but we included only participants aged 40 or older according to the needs of the study. The outcome variables related to lung function that we included in the study included FEV1, FVC and FEV1: FVC. NHANES provides FEV1 and FVC quality ratings [according to the American Thoracic Society (ATS) standard] (38), and we limit our analysis to vital capacity measurements of participants with FEV1 and FVC quality ratings of A (exceeding ATS data collection standards) or B (meeting ATS data collection standards). The ratio of FEV1 to FVC is obtained by dividing FEV1 by FVC. After excluding participants with missing variables and unqualified lung function data, we finally included 6,204 participants in our study (Supplementary Figure 1).

The three respiratory symptoms we examined were dyspnea, cough and expectoration. We also limit these analyses to adults aged 40 and older. Dyspnea was defined according to participants’ answers to the question “shortness of breath on stairs/inclines.” This part of the data exists in all survey cycles. The definition of cough and expectoration is based on the problem of “coughing for more than three months of the year” and “expectoration of sputum for more than three months of the year,” but the data for cough and expectoration are only available in the 2007–2012 survey period. For the inclusion process of this part of the study population, see (Supplementary Figures 2, 3).

#### Exposure

Using data collected by NHANES and the United States Department of Agriculture (USDA), we obtained scores for two healthy eating patterns-the Mediterranean diet and the DASH diet.

#### Assessment of dietary intake

We got diet-related data from the National Health and Nutrition Examination Survey (NHANES) (36) and the United States Department of Agriculture (USDA) (39). It is divided into three parts, including personal food and total nutrient data from NHANES and the Food Patterns Equivalents Database (FPED) from USDA.

All NHANES participants were eligible to participate in two 24-h food recall interviews. The first dietary memory interview was collected in person at the Mobile Test Center (MEC), and the second interview was collected by telephone 3–10 days later.

The purpose of the diet interview section is to obtain detailed dietary intake information from NHANES participants. Dietary intake data are used to estimate the type and quantity of food and beverages (including all types of water) consumed during the 24 h prior to the interview (midnight to midnight), as well as to estimate energy, nutrition and other food components in these foods and drinks.

#### Assessment of dietary scores

Based on data collected by NHANES and USDA, we have scores for two healthy diets recommended in the 2020–2025 Dietary Guidelines for Americans (22). The Diet to stop Hypertension (DASH) and the Mediterranean Diet. Based on the participants’ response to food and nutrient intake, which was emphasized or minimized in the DASH diet, a previously derived DASH diet score was generated for each participant. DASH scores are based on nine target nutrients (saturated fat, total fat, protein, cholesterol, fiber, magnesium, calcium, potassium, and sodium) (40). For each target nutrient, participants who reached the target were given a score of 1, while participants who reached the intermediate goal were given a score of 0.5 (maximum score = 9) (40, 41). The higher the intake of protein, cellulose, magnesium, calcium or potassium, the higher the score, while the lower the intake of saturated fat, total fat, cholesterol or sodium, and the higher the score. Supplementary Table 8 summarizes the dietary variables and their scoring criteria using DASH scores in this article.

We calculated the Mediterranean Diet compliance score using the Mediterranean Diet Index based on literature design constructed by Sofi et al. (42). SOFI compliance scores are derived from nine food categories (fruits, vegetables, legumes, fish, red meat, dairy products, alcohol, and olive oil) assigned values of “0,” “1” or “2.” The higher the score, the better the compliance with the Mediterranean Diet. The higher score of the Mediterranean Diet reflects the higher intake of each food ingredient, but the red meat and alcohol intake. In the Mediterranean Diet scoring system constructed by Sofi et al. these scores were calculated using the gram intake reference for each category, except for the classification reference 0 = never, 1 = sometimes, and 2 = frequently for olive oil. We calculated the Mediterranean Diet score using the FPED grouping from USDA and their reported intake in grams. However, the FPED fruit and vegetable variables were reported as cup equivalent (CE) intake. Therefore, we modified the Mediterranean Diet fruit score to reflect 0 = < 1 CE, 1 = ≥ 1 CE, and 2 = ≥ 2 CE and the Mediterranean Diet vegetable scores to reflect 0 = <0.5 CE, 1 = ≥ 0.5 CE, and 2 = ≥ 1 CE. We also extracted olive oil intake (in grams) from NHANES personal food ingredient intake data and modified the score to reflect 0 = <14 g, 1 = ≥ 14 g, and 2 = ≥ 28 g (43). In addition, the scores of all other the Mediterranean Diet components are calculated according to Sofi et al. Supplementary Table 9 summarizes the dietary variables and their scoring criteria used in this literature.

#### Standard COPD risk factors

National Health and Nutrition Examination Survey provided participants with primary tobacco exposure, secondhand smoke exposure, occupational exposure and asthma. In NHANES, participants’ smoking status (now, ever, and never) was self-reported and confirmed by blood cotinine measurements (44). We defined smoking status according to the answers to two questions: “have you smoked at least 100 cigarettes in your life” and “do you smoke now?” Participants who answered “YES” both questions were defined as current smokers. Participants who smoked at least 100 cigarettes in their lifetime were classified as previous smokers if they did not meet the current definition of smokers. Participants who smoked less than 100 cigarettes in their lifetime were classified as non-smokers. The number of pack-years is calculated by multiplying the number of years of smoking by the average number of cigarettes smoked per day divided by 20.

National Health and Nutrition Examination Survey also reported data on household smokers, based on which we assessed participants’ exposure to secondhand smoke. Due to the lack of data, we may have ignored the exposure to secondhand smoke at work.

Only 2007–2012 cycles in the NHANES assess occupational exposure to steam, dust or smoke and the number of years of occupational exposure. Therefore, we use these data to discuss the impact of occupational exposure on the model in the sensitivity analysis part. If the participants are exposed to both dust and steam or smoke, we define the occupational exposure years as the sum of these exposure types.

We define asthma as having been diagnosed by a doctor or health professional.

#### Other variables

Gender, age, race/ethnicity, marital status, education, and family income data are from the demographic data section of NHANES. Body mass index (BMI) data are from body measurement section in the examination data of NHANES. In addition, we convert age into classified data according to the characteristics of the data and statistical needs (45), because this can make a better subgroup analysis in age. In the original data, marital status is six-classified data, we also convert cohabitation into two-classified data. We also make appropriate adjustments to education, family income-poverty ratio and BMI.

### Statistical analysis

The characteristic analysis, univariate analysis and multivariate analysis of the participants in this study are all weighted according to the reverse probability based on population selection to provide estimates of the population of the United States (46).

A primary analysis assessed the relationship between dietary patterns (including DASH diet and Mediterranean diet) and COPD, and we also analyzed the relationship between dietary patterns and pulmonary function (FEV1: FVC) and respiratory symptoms (dyspnea, cough, and expectoration). Secondary analysis explored the relationship between dietary patterns and COPD stratified according to age, sex and smoking history, and subgroup analysis on the relationship between dietary patterns and pulmonary function or respiratory symptoms in COPD and non-COPD populations.

Logistic regression model was used to evaluate the relationship between dietary patterns and COPD prevalence. The DASH score and the Mediterranean score were analyzed both as a categorical variable (tertiles). The regression model was not adjusted at first, and then adjusted gradually according to (1) age, sex, race/ethnicity, cohabitation, education, family income-poverty ratio, BMI, and total energy intake; (2) primary tobacco smoke exposure (smoking status and pack-years); and (3) secondhand smoke exposure and asthma. Linear regression model was used to evaluate the relationship between dietary patterns and FEV1: FVC, and the above variables were adjusted. We also used logistic regression models to evaluate the relationship between diet and respiratory symptoms. In addition, we use the likelihood ratio test to test the significance of the interaction.

We analyzed the sensitivity from the following three aspects: (1) Adding occupational exposure to the final model; (2) replacing FEV1: FVC with FEV1 or FVC; and (3) excluding people with cardiovascular disease, cancer or diabetes. Analyses were performed using R (version 4.0.4). In order to illustrate the complex survey design and the representative estimate of the population of the United States, the survey package was used for analysis. The significance threshold was 0.05 and the bilateral *P*-value was reported.

## Results

This study included 2,488 COPD participants and 25,607 non-COPD participants. We found that the age of COPD participants was older than that of non-COPD participants (60.2 vs. 56.9) and the proportion of women in COPD participants was higher than that of non-COPD participants (63.7 vs. 51.4%). The BMI was similar between participants with COPD and participants with non-COPD (29.1 vs. 30.5). The proportion of non-Hispanic whites in COPD group was significantly higher than that in non-COPD group (80.9 vs. 74.0). In addition, COPD participants had lower rates of cohabitation (57.5 vs. 69.5%), education [49.0 vs. 59.5% (More than high school)], family income-poverty ratio (2.6 vs. 3.3), and total dietary energy (1919.30 vs. 2054.31). More importantly, COPD participants had higher rates of smoking [32.9 vs. 16.9% (current smokers)], secondhand smoke exposure (34.4 vs. 18.5%), and asthma (42.6 vs. 10.1%). In addition, Mediterranean diet scores based on nine dietary components and DASH diet scores based on nine nutrients were lower in participants with COPD [5.59 vs. 5.81 (Mediterranean diet scores); 2.25 vs. 2.51 (DASH diet scores)] (Table 1).

**TABLE 1:** Basic characteristics of the study participants.

	Participants without COPD (unweighted *n* = 25,607; weighted *n* = 104,188,168)			Participants with COPD (unweighted *n* = 2,488; weighted *n* = 101,762,55)			*P* ^a^
**Characteristic**	***N***	**W%**	**CI (95%)**	***N***	**W%**	**CI (95%)**	
Gender							<0.001
Male	12,828	48.6	48.0−49.2	1,017	36.3	33.9−38.8	
Female	12,779	51.4	50.8−52.0	1,471	63.7	61.2−66.1	
Age							<0.001
40–50	6,914	32.9	31.9–34.0	466	24.0	21.5−26.6	
50−60	5,948	29.2	28.3−30.1	536	25.6	22.7−28.6	
>60	12,745	37.9	36.8−39.0	1,486	50.4	47.8−53.0	
Mean ± SE	25,607	56.9 ± 0.14		2,488	60.2 ± 0.30		<0.001
Race/ethnicity							<0.001
Non-Hispanic White	12,050	74.0	72.0−76.0	1,558	80.9	78.6−83.1	
Non-Hispanic Black	5,386	10.0	8.9−11.1	432	8.0	6.8−9.4	
Mexican American	4,187	5.9	5.0−6.9	195	2.4	1.8−3.1	
Other Hispanic	2,001	4.4	3.7−5.3	152	2.8	2.1−3.6	
Other race	1,983	5.7	5.2−6.3	151	5.9	4.8−7.3	
Cohabitation							<0.001
No	9,246	30.5	29.5−31.5	1,198	42.5	39.8−45.2	
Yes	16,361	69.5	68.5−70.5	1,290	57.5	54.8−60.2	
Education							<0.001
High school	5,902	24.1	23.2−25.1	622	27.4	25.3−29.5	
Less than high school	7,147	16.4	15.4−17.4	786	23.6	21.2−26.1	
More than high school	12,558	59.5	58.0−61.0	1,080	49.0	46.1−51.2	
Income-poverty ratio							<0.001
≤2	11,084	28.4	26.9−29.9	1,439	45.3	41.8−49.0	
>2	14,523	71.6	70.1−73.1	1,049	54.7	51.0−58.2	
Mean ± SE	25,607	3.3 ± 0.03		2,488	2.6 ± 0.06		<0.001
BMI							<0.001
18.5−25	6,282	25.5	24.6−26.4	564	21.6	19.5−23.7	
<18.5	269	1.0	0.8−1.2	67	2.8	2.1−3.6	
25−30	9,332	36.0	35.1−36.9	718	29.0	26.8−31.3	
>30	9,724	37.5	36.5−38.5	1,139	46.6	44.0−49.3	
Mean ± SE	25,607	29.1 ± 0.07		2,488	30.5 ± 0.19		<0.001
Total energy, kcal/day							
Mean ± SE	25,607	2054.17 ± 7.45		2,488	1919.30 ± 20.54		<0.001
Smoking status							<0.001
Never	13,323	52.6	51.5−53.6	735	30.5	27.9−33.2	
Ever	7,846	30.5	29.7−31.4	979	36.6	34.0−39.3	
Now	4,438	16.9	16.2−17.6	774	32.9	29.8−36.1	
Pack-years							
Mean ± SE	25,607	10.8 ± 0.22		2,488	23.7 ± 0.83		<0.001
Secondhand smoke							<0.001
No	20,659	81.5	80.4−82.6	1,659	65.6	62.2−68.9	
Yes	4,948	18.5	17.4−19.6	829	34.4	31.1−37.8	
Asthma							<0.001
No	23,154	89.9	89.4−90.4	1,423	57.4	54.8−60.0	
Yes	2,453	10.1	9.6−10.6	1,065	42.6	40.0−45.2	
MedD scores							0.002
T1	8,272	32.3	31.4−33.3	915	35.6	32.6−38.6	
T2	8,442	33.0	32.1−33.9	845	34.2	31.7−36.8	
T3	8,893	34.7	33.7−35.7	728	30.2	27.9−32.6	
Mean ± SE	25,607	5.81 ± 0.02		2,488	5.59 ± 0.05		<0.001
DASH scores							<0.001
T1	8,317	32.9	32.0−33.9	982	40.1	37.4−42.8	
T2	8,590	34.7	33.8−35.5	831	33.6	31.4−35.9	
T3	8,700	32.4	31.4−33.3	675	26.3	23.8−28.9	
Mean ± SE	25,607	2.51 ± 0.01		2,488	2.25 ± 0.04		<0.001

CI, confidence interval; BMI, body mass index; MedD, Mediterranean diet; DASH, dietary approaches to stop hypertension.

^a^*P*-values are design-adjusted Rao-Scott Pearson χ2 test for categorical variables and *T*-test for continuity variables.

In addition, Supplementary Table 1 shows the characteristics of participants based on the Mediterranean diet score and the DASH diet score. We found that people with the highest scores on the Mediterranean diet had fewer women, older, more whites, higher socioeconomic status, higher total energy intake and were less likely to be exposed to tobacco and secondhand smoke than those with the lowest scores. However, those with the highest scores on DASH diet had fewer women, younger ages and fewer whites than those with the lowest scores on DASH diet. Similar to the Mediterranean score, participants with the highest DASH score had higher socioeconomic status, higher total energy, less current smokers and less likely to be exposed to secondhand smoke than those with the lowest DASH score (Supplementary Table 1).

We first evaluated the relationship between Mediterranean dependence score and COPD. The unadjusted weighted model showed that patients with the highest third of Mediterranean diet score were associated with a lower prevalence of COPD than those with the lowest third of Mediterranean diet score (OR: 0.79; 95% CI: 0.69–0.91; *P* = 0.001). After adjusting all the covariates of interest (model 3), this correlation no longer exists (OR: 1.03; 95% CI: 0.88–1.20; *P* = 0.697). In contrast, both the pre-adjusted model and the post-adjusted model had a lower prevalence of COPD among the participants with the highest dietary compliance than those with the lowest compliance [OR: 0.67; 95% CI: 0.57–0.77; *P* < 0.001 (Unadjusted model); OR: 0.74; 95% CI: 0.63–0.86; *P* < 0.001 (model 1); OR: 0.82; 95% CI: 0.70–0.95; *P* = 0.011 (model 2); OR: 0.83; 95% CI: 0.71–0.97; *P* = 0.021 (model 3)] (Table 2).

**TABLE 2:** Associations of adherence to DASH and Mediterranean diet with COPD.

Adjusted model												
Characteristic	Unadjusted model			Model 1^a^			Model 2^b^			Model 3^c^		
	OR	CI (95%)	*P*-value	OR	CI (95%)	*P*-value	OR	CI (95%)	*P*-value	OR	CI (95%)	*P*-value
**MedD scores**												
T1	Ref			Ref			Ref			Ref		
T2	0.94	0.81−1.08	0.400	0.89	0.77−1.03	0.124	0.96	0.83−1.11	0.603	1.06	0.91−1.23	0.452
T3	0.79	0.69−0.91	0.001	0.80	0.69−0.92	0.003	0.95	0.81−1.10	0.457	1.03	0.88−1.20	0.697
**DASH scores**												
T1	Ref			Ref			Ref			Ref		
T2	0.80	0.71−0.90	<0.001	0.81	0.72−0.91	0.001	0.85	0.76−0.96	0.010	0.83	0.73−0.95	0.005
T3	0.67	0.57−0.77	<0.001	0.74	0.63−0.86	<0.001	0.82	0.70−0.95	0.011	0.83	0.71−0.97	0.021

CI, confidence interval; OR, odds ratio; MedD, Mediterranean diet; DASH, dietary approaches to stop hypertension.

^a^Adjusted for gender, age, race/ethnicity, cohabitation, education, income-poverty ratio, body mass index, and total energy.

^b^Adjusted for gender, age, race/ethnicity, cohabitation, education, income-poverty ratio, body mass index, total energy, smoking, and pack-years.

^c^Adjusted for gender, age, race/ethnicity, cohabitation, education, income-poverty ratio, body mass index, total energy, smoking, pack-years, secondhand smoke, and asthma.

Although we did not observe the interaction between DASH diet (interaction *P*-value is 0.909) or Mediterranean diet (interaction *P*-value is 0.245) and smoking status, we conducted a stratified analysis according to smoking status, because smoking is a major risk factor for the development of COPD. When stratifying participants according to smoking status, we found that the association between higher compliance of DASH diet and lower risk of COPD still existed in participants with a history of smoking (OR: 0.82; 95% CI: 0.68–0.98; *P* = 0.033). However, we did not find a correlation between Mediterranean dietary compliance and the prevalence of COPD in a stratified analysis of smoking history (OR: 1.00; 95% CI: 0.84–1.21; *P* = 0.963). In addition, we conducted a stratified analysis of age and sex and found that the relationship between DASH diet and COPD still existed in men (OR: 0.73; 95% CI: 0.58–0.93; *P* = 0.010) and relatively young participants (OR: 0.74; 95% CI: 0.55–1.01; *P* = 0.055) (Table 3).

**TABLE 3:** Subgroup analysis of gender, age and smoking status about associations of adherence to DASH and Mediterranean diet with COPD.

Adjusted model													
Characteristic	Unadjusted model			Model 1^a^			Model 2^b^			Model 3^c^			*P* for interaction
	OR	CI (95%)	*P*-value	OR	CI (95%)	*P*-value	OR	CI (95%)	*P*-value	OR	CI (95%)	*P*-value	
**MedD scores**													
Gender													0.001
Male	0.84	0.68−1.03	0.087	0.89	0.72−1.11	0.310	1.07	0.84−1.36	0.577	1.12	0.88−1.44	0.358	
Female	0.69	0.59−0.81	<0.001	0.73	0.62−0.87	<0.001	0.87	0.73−1.05	0.141	0.97	0.80−1.17	0.742	
Age													0.731
40−50	0.69	0.51−0.95	0.022	0.74	0.53−1.03	0.070	0.86	0.62−1.20	0.380	0.92	0.65−1.30	0.629	
50−60	0.84	0.63−1.11	0.210	0.94	0.71−1.25	0.679	1.18	0.89−1.56	0.241	1.23	0.92−1.67	0.166	
>60	0.77	0.65−0.91	0.003	0.78	0.65−0.94	0.007	0.92	0.76−1.12	0.419	1.02	0.83−1.25	0.839	
Smoking status													0.245
No	1.02	0.79−1.32	0.876	1.03	0.78−1.37	0.816	NA			1.08	0.81−1.45	0.577	
Yes	0.84	0.71−1.00	0.047	0.84	0.70−1.00	0.052	0.87	0.73−1.05	0.157	1.00	0.84−1.21	0.963	
**DASH scores**													
Gender													0.317
Male	0.64	0.52−0.79	<0.001	0.69	0.55−0.87	0.002	0.76	0.60−0.95	0.019	0.73	0.58−0.93	0.010	
Female	0.72	0.59−0.88	0.002	0.77	0.63−0.95	0.014	0.86	0.70−1.06	0.158	0.91	0.73−1.13	0.375	
Age													0.598
40−50	0.61	0.46−0.80	<0.001	0.71	0.53−0.96	0.027	0.74	0.55−1.00	0.052	0.74	0.54−1.01	0.055	
50−60	0.72	0.54−0.96	0.024	0.82	0.60−1.12	0.203	0.92	0.67−1.26	0.603	0.92	0.68−1.26	0.614	
>60	0.70	0.57−0.85	0.001	0.73	0.59−0.90	0.003	0.82	0.66−1.01	0.066	0.85	0.68−1.06	0.155	
Smoking status													0.909
No	0.73	0.56−0.95	0.021	0.83	0.63−1.10	0.186	NA			0.87	0.65−1.16	0.339	
Yes	0.68	0.58−0.81	<0.001	0.77	0.64−0.91	0.003	0.79	0.66−0.94	0.009	0.82	0.68−0.98	0.033	

CI, confidence interval; OR, odds ratio; MedD, Mediterranean diet; DASH, dietary approaches to stop hypertension.

^a^Adjusted for gender, age, race/ethnicity, cohabitation, education, income-poverty ratio, body mass index, and total energy.

^b^Adjusted for gender, age, race/ethnicity, cohabitation, education, income-poverty ratio, body mass index, total energy, smoking, and pack-years.

^c^Adjusted for gender, age, race/ethnicity, cohabitation, education, income-poverty ratio, body mass index, total energy, smoking, pack-years, secondhand smoke, and asthma.

We also analyzed the relationship between DASH diet or Mediterranean diet and secondary outcome events (Table 4). We found that participants’ risk of FEV1: FVC decrease, dyspnea, cough or expectoration was negatively correlated with DASH dietary compliance [β: −0.01; 95% CI: −0.01–0.00; *P* = 0.003 (FEV1: FVC); OR: 0.80; 95% CI: 0.73–0.89; *P* < 0.001 (dyspnea); OR: 0.80; 95% CI: 0.70–0.91; *P* = 0.002; OR: 0.86; 95% CI: 0.74–0.99; *P* = 0.042). However, Mediterranean dietary compliance was only negatively correlated with the risk of cough (OR: 0.83; 95% CI: 0.72–0.97; *P* = 0.021). In addition, we analyzed the relationship between dietary compliance and secondary outcome events in COPD and non-COPD populations (Supplementary Table 2). In particular, we found that there was a negative correlation between DASH compliance and FEV1: FVC in non-COPD population (β: −0.01; 95% CI: −0.01–0.00; *P* = 0.011), but this relationship did not exist in COPD population.

**TABLE 4:** Associations of adherence to DASH and Mediterranean diet with secondary outcome variables.

Characteristic	MedD scores			DASH scores		
FEV1/FVC	β	CI (95%)	*P*-value	β	CI (95%)	*P*-value
Unadjusted model	0.01	0.00−0.02	0.006	−0.01	−0.01−0.00	0.053
a	0.01	0.01−0.02	0.002	0.00	−0.01−0.00	0.173
b	0.00	0.00−0.01	0.271	−0.01	−0.01−0.00	0.005
c	0.00	0.00−0.01	0.355	−0.01	−0.01−0.00	0.003
**Dyspnea**	**OR**	**CI (95%)**	***P*-value**	**OR**	**CI (95%)**	***P*-value**
Unadjusted model	0.78	0.72−0.85	<0.001	0.66	0.61−0.73	<0.001
a	0.80	0.73−0.88	<0.001	0.75	0.68−0.83	<0.001
b	0.89	0.81−0.98	0.020	0.80	0.72−0.88	<0.001
c	0.92	0.84−1.01	0.088	0.80	0.73−0.89	<0.001
**Cough**	**OR**	**CI (95%)**	***P*-value**	**OR**	**CI (95%)**	***P*-value**
Unadjusted model	0.62	0.54−0.71	<0.001	0.65	0.56−0.74	<0.001
a	0.63	0.54−0.73	<0.001	0.68	0.59−0.78	<0.001
b	0.82	0.70−0.95	0.009	0.80	0.70−0.92	0.003
c	0.83	0.72−0.97	0.021	0.80	0.70−0.91	0.002
**Expectoration**	**OR**	**CI (95%)**	***P*-value**	**OR**	**CI (95%)**	***P*-value**
Unadjusted model	0.75	0.65−0.88	<0.001	0.74	0.64−0.85	<0.001
a	0.77	0.66−0.91	0.002	0.73	0.63−0.85	<0.001
b	1.01	0.84−1.20	0.949	0.86	0.74−1.01	0.059
c	1.03	0.84−1.23	0.744	0.86	0.74−0.99	0.042

CI, confidence interval; OR, Odds Ratio; MedD, Mediterranean diet; DASH, dietary approaches to stop hypertension.

^a^Adjusted for gender, age, race/ethnicity, cohabitation, education, income-poverty ratio, body mass index, and total energy.

^b^Adjusted for gender, age, race/ethnicity, cohabitation, education, income-poverty ratio, body mass index, total energy, smoking, and pack-years.

^c^Adjusted for gender, age, race/ethnicity, cohabitation, education, income-poverty ratio, body mass index, total energy, smoking, pack-years, secondhand smoke, and asthma.

After further adjusting occupational exposure, we have come to the same conclusion (Supplementary Table 3). The relationship between the DASH diet and COPD persisted after excluding participants with cardiovascular disease, cancer, or diabetes (Supplementary Table 4). We also found that there was a negative correlation between DASH dietary compliance and FVC, which still existed in non-COPD population (Supplementary Tables 5, 6).

## Discussion

Among American adults, we found that dietary patterns with higher DASH dietary scores, which reflecting the nutrients (saturated fat, total fat, protein, cholesterol, fiber, magnesium, calcium, potassium, and sodium) were associated with a lower risk of COPD. This association still exists in several subgroups (male, relatively young, and smoker). In contrast, in this large cross-sectional analysis representing the adult population of the United States, there was no significant correlation between Mediterranean dietary score and COPD prevalence. In addition, we also found associations between DASH dietary compliance and lung function or respiratory symptoms, especially in non-COPD populations. Taken together, these findings expand the association between DASH diet and chronic diseases and support the importance of diet in the pathogenesis of COPD.

The earliest literature on the relationship between diet and COPD focused on individual nutrients and selected foods. For example, some studies have investigated the relationship between nutrients (such as vitamin C, vitamin E, omega-3 fatty acids, and flavonoids) and COPD and obtained positive results (47–50). Other studies have shown that intake of fruits and vegetables can reduce the incidence of COPD and improve lung function (14, 51, 52). But some studies have shown that individual nutrients or selected foods have no significant effect on COPD or lung function (19–21). In this case, dietary patterns provide an overview of diet and are a good way to assess the relationship between diet and COPD. Previous studies have explored the relationship between dietary patterns and COPD, including Western diet, traditional diet, prudent diet, Mediterranean diet, healthy eating index and so on.

A total of 11 studies investigated the relationship between dietary patterns and vital capacity measurement or COPD, including four from the United States (12, 13, 53, 54) and seven from other countries (55–61). Four studies from the United States explored the effects of Western diet, prudent diet and healthy eating index on COPD. Three of the four studies reported a “protective” association with a “cautious” diet, characterized by high intake of fruits, vegetables, fish and whole grains, consistent with the dietary antioxidant or anti-inflammatory hypothesis. In addition, the three studies also reported the harmful effects of the “Western” diet. One of the four studies also found the importance of healthy eating index for COPD. Our study explored the relationship between the dietary patterns recommended by the guidelines (DASH diet and the Mediterranean diet) and COPD.

Dietary Approaches to Stop Hypertension diet trial is a multicenter randomized feeding study that tests the effects of dietary patterns on blood pressure (62). Then it was gradually discovered that this diet has a protective effect on cardiovascular disease (63–65), cancer (30, 66, 67), and metabolic diseases (29, 31, 32, 68, 69). However, only one case-control study from Iran explored the relationship between DASH diet and COPD, and they found significant differences in DASH diet between the case group and the control group (61). To the best of our knowledge, our study is the first to investigate the association between DASH diet and the risk of COPD in the adult population of the United States.

Three European studies have explored the role of the Mediterranean diet in the development of COPD (55–57). A cross-sectional study from Spain did not find the effect of the Mediterranean diet on lung function in smokers without respiratory disease (55). However, another study from Spain found that the Mediterranean diet was associated with better lung function in people without lung disease (56). A recent case-control study from Sweden also confirmed the protective effect of the Mediterranean diet on COPD (57). Our research shows that the Mediterranean diet does not affect the occurrence of COPD in the American population. This phenomenon suggests that regional differences in diet may affect the role of diet in the development of COPD. The Mediterranean diet score was also based on the Mediterranean population, especially Italians, so the Mediterranean diet score does not represent the eating habits of the American population. And the American population has a low level of adherence to the Mediterranean diet, with an average score of 5.8 (the highest score is 18). So we have not concluded that the Mediterranean diet can affect the prevalence of COPD in the American population.

Oxidative stress and related inflammation in lungs and circulation are the main pathogenic processes of COPD after long-term exposure to risk factors such as tobacco smoke and air pollution (70). Diet may also contribute to oxidation and inflammation in patients with COPD (9). Therefore, we speculate that the antioxidants and anti-inflammatory components in the DASH diet may prevent or delay the occurrence of COPD. Another possible mechanism is the influence of intestinal microbiota (71). Studies have shown that dietary fiber can lead to changes in the composition of intestinal and lung microflora, and mice fed a high-fiber diet for a long time can protect themselves from allergic inflammation of the lungs (72). The lack of fermentable fiber may lead to microflora malnutrition, cause intestinal biological disorders and affect lung physiology (73). The third mechanism is that advanced glycation end products (AGEs) may affect the occurrence and development of COPD through inflammation (74). In addition, the AGEs in many diets has high molecular weight and is not absorbed in the intestines, but enters the colon, where they can be metabolized by colon bacteria (75). Therefore, AGEs has the same effect on the composition of intestinal microflora and beneficial microbial metabolites. This may suggest the existence of the AGEs- gut-lung axis, whose core is the imbalance between oxidation and inflammation.

We also found that the DASH diet can affect lung function, especially in non-COPD participants. In addition, the effect of DASH diet on the prevalence of COPD was more significant in relatively young people. These suggest that DASH diet has a greater impact on lung function when there is no significant decline in lung function, that is to say, we should prevent the occurrence of COPD and delay the decline of lung function through diet earlier. Although the DASH diet had little effect on lung function that had been significantly decreased, the DASH diet improved respiratory symptoms, regardless of whether or not the COPD population. In the subgroup analysis of smokers and non-smokers, we only found a significant correlation between DASH diet and COPD in smokers. This may be because smoking causes severe oxidative and inflammatory exposure to the lungs, and the DASH diet can improve the adverse effects of smoking. In addition, we believe that men have higher levels of smoking and oxidative stress, which may explain why men are generally more affected by the DASH diet than women.

The analysis was conducted using data from NHANES, a nationally representative health and nutrition survey in the United States. The study is a sample survey design that allows overall estimates based on a high level of confidence and represents the entire population of the United States (76). The advantages of this analysis include a large sample size (*n* = 28,095) and contain a wealth of information about lifestyle, socio-economic and environmental factors, and lung function data are obtained in the context of rigorous spirometry programs conducted by trained field staff. In addition, our study of dietary patterns, rather than individual foods or nutrients, helps to address the effects of eating habits in the context of their lifestyles. Dietary pattern analysis takes into account the interaction between nutrients, so the effect of the whole diet can be taken into account. Understanding the impact of dietary patterns can help develop preventive measures to stimulate changes in the eating habits of specific populations.

The study also has several limitations. First, the cross-sectional nature of this analysis does not allow the evaluation of causality, and we cannot rule out the possibility that poor living habits (such as smoking) of COPD people affect food decision-making, resulting in a low score of dietary compliance. However, “reverse causality” does not seem to be a possible explanation for the main findings, because our participants with COPD seem less likely to choose an unhealthy diet. Moreover, after excluding the interference of many confounding factors, the results are still robust. Many prospective studies have also supported the relationship between dietary patterns and COPD (41, 42, 44). Second, the assessment of 24-h dietary intake may not accurately reflect the diet of individual participants in their lifetime, that is, whether the reported intake truly reflects the actual daily dietary intake. However, 24-h dietary recall is widely considered to be the most appropriate dietary assessment method for estimating the average intake of the population (67). Its open nature allows for the most specific details about the foods and recipes consumed. In addition, we took the average of two 24-h diet recall interviews to reduce this error. Third, the diagnosis of COPD was self-reported by participants as to whether they had been told by a doctor or other health specialist that they had chronic bronchitis and emphysema, as only a limited number of years (2007–2012) had lung function measurements. However, we also found a correlation between the DASH diet and lung function using data from these years. Fourth, we acknowledge that the link between DASH diet and COPD may be partly due to residual confusion caused by smoking, which is a powerful risk factor. In order to minimize this possibility, we adjusted the regression model according to primary tobacco exposure and secondhand smoke exposure. Even after we controlled for all these factors, the negative correlation between DASH diet and COPD risk still existed. Fifth, the differences between excluded missing data and participants included in the study may lead to bias and limit generality. This is unlikely considering that the inclusion excludes the similarity between the baseline characteristics of participants, especially the standard COPD risk factors (Supplementary Table 7). Sixth, due to the lack of data in some years, we cannot adjust occupational dust exposure, which is still an important risk factor for COPD. However, we included occupational dust exposure as a confounding factor in the final model in the 2007–2012 data, and DASH diet can also affect the prevalence of COPD (DASH diet as a continuous variable, *P* = 0.037). Seventh, Considering the known associations between diet and cardiovascular disease, tumors, and diabetes, we reanalyzed in a population that excluded these participants and found an association between the DASH diet and COPD.

## Conclusion

In conclusion, DASH dietary score was associated with the prevalence of COPD, lung function and respiratory symptoms. This new finding supports the importance of diet in the pathogenesis of COPD and expands the scope of the correlation between DASH diet and major chronic diseases. Although efforts to prevent COPD should continue to focus on quitting smoking, these findings support the importance of healthy eating in a number of intervention programs to prevent COPD. Our results encourage clinicians to consider the potential role of the combined effects of food in a healthy diet in promoting lung health. More prospective and clinical trial interventions are needed in the future to confirm the effectiveness of DASH diet in the prevention of respiratory disease.

## Data availability statement

The raw data supporting the conclusions of this article will be made available by the authors, without undue reservation.

## Ethics statement

The studies involving human participants were reviewed and approved by the Research Ethics Review Board of the National Centre for Health Statistics. The patients/participants provided their written informed consent to participate in this study.

## Author contributions

XQ had full access to all the data in the study and took responsibility for the integrity of the data and the accuracy of the data analysis, did critical revision of the manuscript for important intellectual content, and obtained funding. JW and XQ performed the concept, design, and supervision of the study. JW, XQ, SG, and XW performed the acquisition, analysis, interpretation of data, administrative, technical, and material support. JW drafted of the manuscript. JW, SG, and XW performed the statistical analysis. All authors contributed to the article and approved the submitted version.
