# Supplementary material for: Associations of adherence to the DASH diet and the Mediterranean diet with chronic obstructive pulmonary disease among US adults
Source: Front Nutr. 2023 Feb 2;10:1031071. doi: 10.3389/fnut.2023.1031071 (PMC9932199; doi:10.3389/fnut.2023.1031071)
Supplement: Supplementary file 1 [file Table_1.docx]

**Supplemental Online Content**

**eFigure 1.** Flow diagram of study participants exploring the association between diet and lung function.

**eFigure 2.** Flow diagram of study participants exploring the association between diet and dyspnea.

**eFigure 3.** Flow diagram of study participants exploring the association between diet and cough or expectoration.

**eTable 1.** Basic characteristics of the study participants by DASH scores and Mediterranean diet scores.

**eTable 2.** Associations of adherence to DASH and Mediterranean diet with secondary outcome variables among participants without COPD and participants with COPD.

**eTable 3.** DASH scores and Mediterranean diet scores associations with COPD with additional adjustment for occupational exposure.

**eTable 4.** Associations of adherence to DASH and Mediterranean diet with COPD after excluding participants in cardiovascular disease, cancer or diabetes.

**eTable 5.** Associations of adherence to DASH and Mediterranean diet with FEV1 and FVC.

**eTable 6.** Associations of adherence to DASH and Mediterranean diet with FEV1 and FVC among participants without COPD and participants with COPD.

**eTable 7.** Characteristics of participants with and without complete data**.**

**eTable 8.** DASH diet composition and scoring criteria.

**eTable 9.** Mediterranean diet composition and scoring criteria.

**
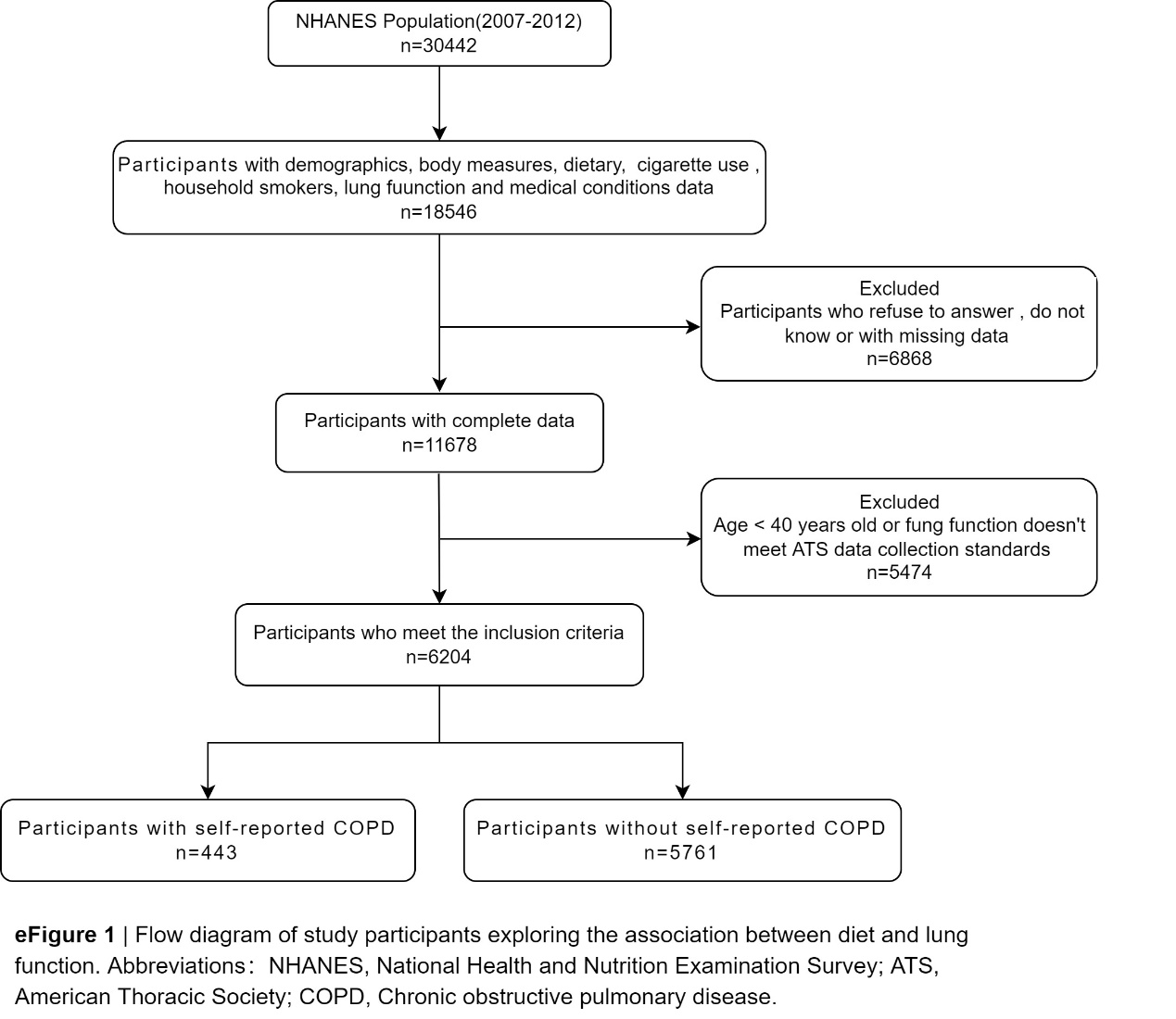
**

**
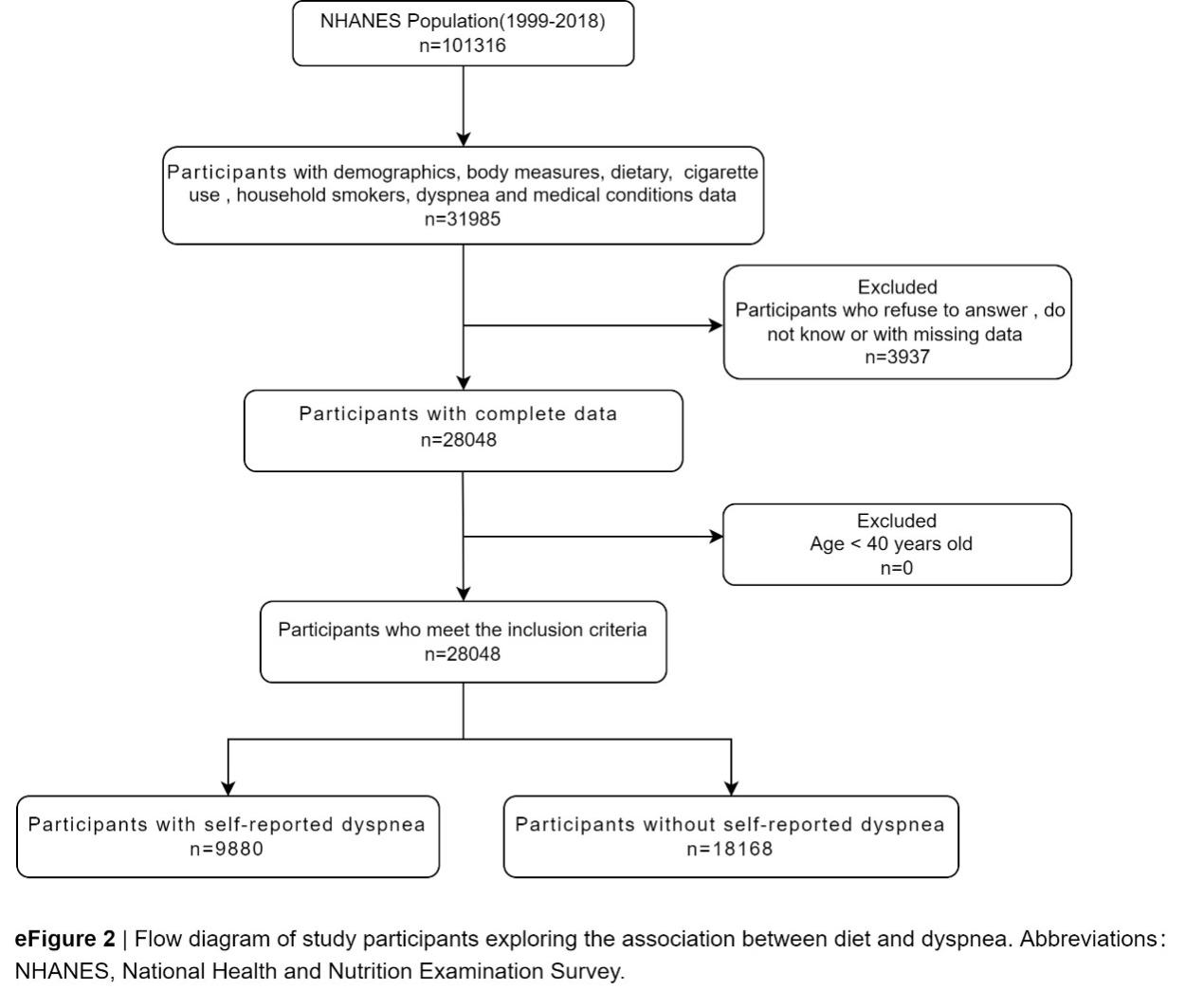
**

**
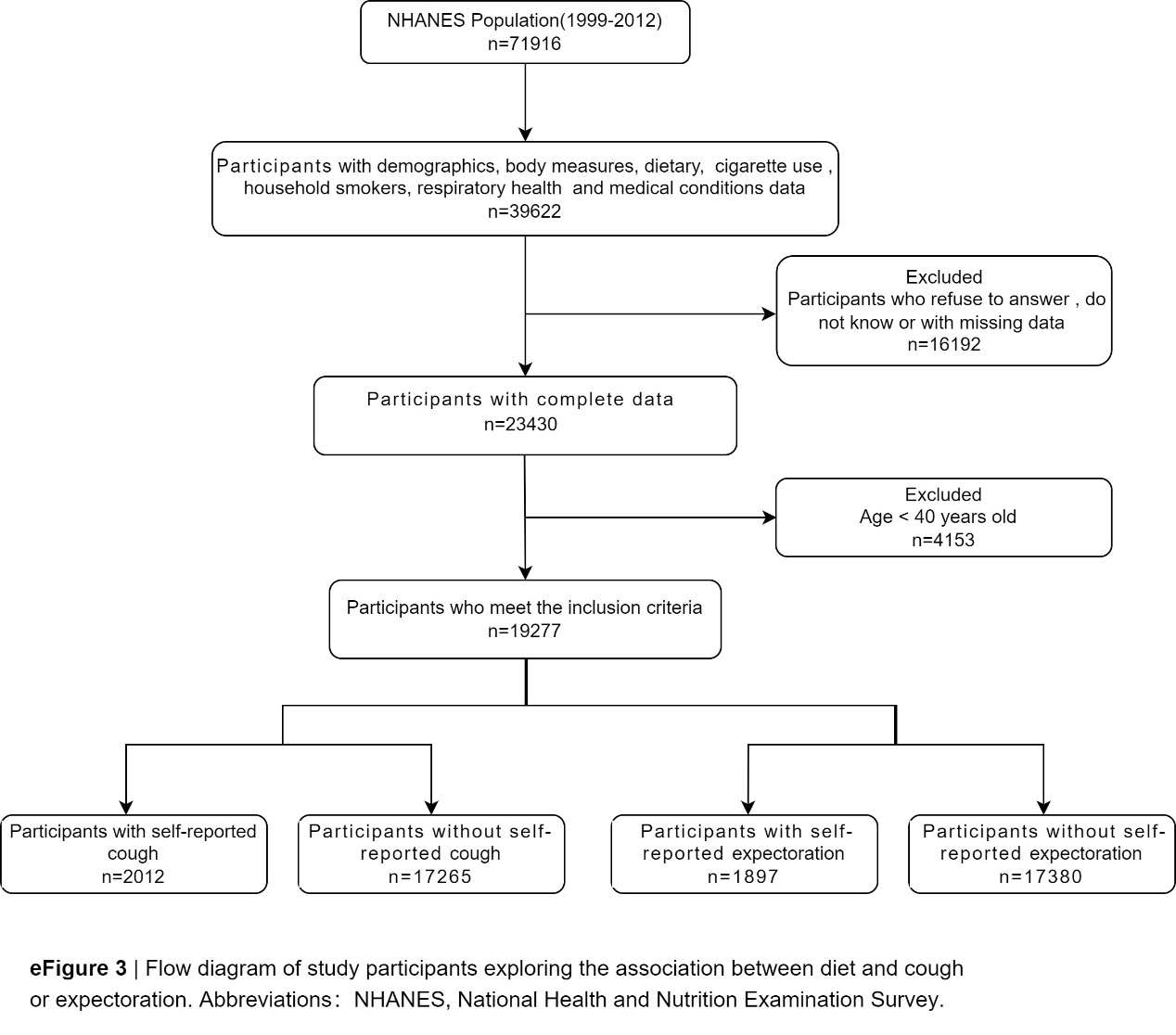
**

**eTable 1. Basic characteristics of the study participants by DASH scores and Mediterranean dietary scores.**

|  | **Mediterranean Diet Adherence** | | | | **DASH Diet Adherence** | | | |
| --- | --- | --- | --- | --- | --- | --- | --- | --- |
| **Characteristic** | **T1** | **T2** | **T3** | ***P* ^a^** | **T1** | **T2** | **T3** | ***P* ^a^** |
| Gender |  |  |  | <.001 |  |  |  | <.001 |
| Male | 54.1 | 45.0 | 43.7 |  | 45.2 | 45.5 | 52.1 |  |
| Female | 45.9 | 55.0 | 56.3 |  | 54.8 | 54.5 | 47.9 |  |
| Age |  |  |  | <.001 |  |  |  | 0.007 |
| 40-50 | 34.5 | 32.2 | 29.9 |  | 31.5 | 31.7 | 33.4 |  |
| 50-60 | 29.5 | 28.2 | 28.9 |  | 27.7 | 29.6 | 29.3 |  |
| >60 | 36.0 | 39.6 | 41.3 |  | 40.8 | 38.8 | 37.3 |  |
| Mean ± SE | 56.3 | 57.3 | 58.0 |  | 57.5 | 57.2 | 56.9 |  |
| Race/ethnicity |  |  |  | <.001 |  |  |  | <.001 |
| Non-Hispanic White | 72.0 | 75.8 | 76.0 |  | 74.9 | 76.7 | 72.1 |  |
| Non-Hispanic Black | 13.2 | 9.7 | 6.7 |  | 12.3 | 9.2 | 7.8 |  |
| Mexican American | 5.2 | 5.2 | 6.2 |  | 4.6 | 5.2 | 7.0 |  |
| Other Hispanic | 4.0 | 4.0 | 4.8 |  | 3.4 | 3.8 | 5.8 |  |
| Other Race | 5.6 | 5.2 | 6.3 |  | 4.8 | 5.2 | 7.3 |  |
| Cohabitation |  |  |  | <.001 |  |  |  | 0.027 |
| No | 33.0 | 32.4 | 29.4 |  | 32.9 | 31.3 | 30.4 |  |
| Yes | 67.0 | 67.6 | 70.6 |  | 67.1 | 68.7 | 69.6 |  |
| Education |  |  |  | <.001 |  |  |  | <.001 |
| High school | 27.3 | 25.8 | 20.4 |  | 28.4 | 24.0 | 20.8 |  |
| Less than high school | 18.3 | 18.2 | 14.6 |  | 16.7 | 16.6 | 17.7 |  |
| More than high school | 54.5 | 56.0 | 65.0 |  | 54.9 | 59.4 | 61.5 |  |
| Income-poverty ratio |  |  |  | <.001 |  |  |  | 0.558 |
| ≤ 2 | 32.8 | 30.8 | 26.1 |  | 29.3 | 30.1 | 30.2 |  |
| ＞2 | 67.2 | 69.2 | 73.9 |  | 70.7 | 69.9 | 69.8 |  |
| Mean ± SE | 3.09 | 3.15 | 3.38 |  | 3.17 | 3.23 | 3.23 |  |
| BMI |  |  |  | <.001 |  |  |  | <.001 |
| 18.5-25 | 23.2 | 23.7 | 28.5 |  | 21.1 | 25.0 | 29.7 |  |
| <18.5 | 1.1 | 1.2 | 1.2 |  | 1.1 | 1.1 | 1.3 |  |
| 25-30 | 34.6 | 35.7 | 35.7 |  | 34.8 | 34.3 | 37.1 |  |
| >30 | 41.1 | 39.4 | 34.6 |  | 43.0 | 39.7 | 31.9 |  |
| Mean ± SE | 29.5 | 29.5 | 28.8 |  | 30.0 | 29.4 | 28.3 |  |
| Total energy, kcal/day |  |  |  | <.001 |  |  |  | <.001 |
| Mean | 1988.33 | 1983.08 | 2150.56 |  | 1999.07 | 1987.15 | 2147.41 |  |
| Smoking status |  |  |  |  |  |  |  |  |
| Never | 42.9 | 50.2 | 58.3 |  | 47.8 | 51.1 | 53.0 |  |
| Ever | 31.2 | 31.2 | 30.9 |  | 31.1 | 30.4 | 31.8 |  |
| Now | 25.8 | 18.6 | 10.8 |  | 21.1 | 18.5 | 15.1 |  |
| Pack-years |  |  |  | <.001 |  |  |  | <.001 |
| Mean | 14.7 | 12.1 | 9.1 |  | 13.6 | 11.5 | 10.5 |  |
| Secondhand Smoke |  |  |  | <.001 |  |  |  | <.001 |
| No | 71.6 | 80.4 | 87.9 |  | 76.3 | 79.8 | 84.3 |  |
| Yes | 28.4 | 19.6 | 12.1 |  | 23.7 | 20.2 | 15.7 |  |
| Asthma |  |  |  | 0.001 |  |  |  | 0.005 |
| No | 85.6 | 87.8 | 87.7 |  | 86.6 | 86.2 | 88.4 |  |
| Yes | 14.4 | 12.2 | 12.3 |  | 13.4 | 13.8 | 11.6 |  |
| MedD scores |  |  |  | <.001 |  |  |  | <.001 |
| Mean | 3.65 | 5.68 | 7.93 |  | 5.16 | 5.65 | 6.60 |  |
| DASH scores |  |  |  | <.001 |  |  |  | <.001 |
| Mean | 2.05 | 2.34 | 3.05 |  | 1.06 | 2.42 | 4.07 |  |

Abbreviations: BMI, body mass index; MedD, mediterranean diet; DASH, dietary approaches to stop hypertension.

^a^ P values are design-adjusted Rao-Scott Pearson χ2 test for categorical variables and T test for continuity variables.

**eTable 2.** Associations of adherence to DASH and Mediterranean diet with secondary outcome variables among participants without COPD and participants with COPD.

|  | **Participants without COPD** | | | **Participants with COPD** | | |
| --- | --- | --- | --- | --- | --- | --- |
| **Characteristic** | **OR/β** | **CI (95%)** | ***P*** | **OR/β** | **CI (95%)** | ***P*** |
| **MedD scores** |  |  |  |  |  |  |
| FEV1/FVC | 0.00 | -0.01 to 0.01 | 0.619 | 0.02 | -0.01 to 0.05 | 0.195 |
| Dyspnea | 0.93 | 0.84-1.02 | 0.109 | 0.90 | 0.68-1.20 | 0.469 |
| Cough | 0.91 | 0.76-1.08 | 0.259 | 0.61 | 0.44-0.85 | 0.004 |
| Phlegm | 1.05 | 0.86-1.28 | 0.605 | 0.98 | 0.70-1.37 | 0.891 |
| **DASH scores** |  |  |  |  |  |  |
| FEV1/FVC | **-**0.01 | -0.01 to 0.00 | 0.011 | -0.01 | -0.03 to 0.01 | 0.399 |
| Dyspnea | 0.83 | 0.75-0.91 | <.001 | 0.71 | 0.52-0.96 | 0.028 |
| Cough | 0.85 | 0.73-1.00 | 0.047 | 0.71 | 0.51-0.98 | 0.038 |
| Phlegm | 0.88 | 0.72-1.07 | 0.194 | 0.93 | 0.66-1.28 | 0.642 |

Abbreviations: CI, confidence interval; OR, Odds Ratio; MedD, mediterranean diet; DASH, dietary approaches to stop hypertension; FEV1, Forced expiratory volume in the first second; FVC, Forced vital capacity.

Logistic regression model was used to evaluate the relationship between dietary patterns and respiratory symptoms (dyspnea, cough, and expectoration) and OR is reported. Linear regression model was used to evaluate the relationship between dietary patterns and FEV1: FVC and β coefficient is reported. All OR and β coefficient in this table are adjusted for gender, age, race/ethnicity, cohabitation, education, income-poverty ratio, body mass index, total energy, smoking, pack-years, secondhand smoke and asthma.

**eTable 3.** DASH scores and Mediterranean diet scores associations with COPD with additional adjustment for occupational exposure.

|  | **Classified variable** | | | **Continuity variable** | | |
| --- | --- | --- | --- | --- | --- | --- |
| **Characteristic** | **OR** | **CI (95%)** | ***P*** | **OR** | **CI (95%)** | ***P*** |
| **MedD scores** |  |  |  |  |  |  |
| Unadjusted model | 0.72 | 0.59-0.88 | 0.001 | 0.92 | 0.88-0.96 | 0.001 |
| Model 1^a^ | 0.75 | 0.60-0.94 | 0.013 | 0.92 | 0.87-0.96 | 0.006 |
| Model 2^b^ | 0.91 | 0.72-1.15 | 0.423 | 0.97 | 0.92-1.03 | 0.298 |
| Model 3^c^ | 0.93 | 0.70-1.23 | 0.595 | 0.97 | 0.91-1.03 | 0.269 |
| Model 4^d^ | 0.95 | 0.71-1.27 | 0.728 | 0.97 | 0.91-1.04 | 0.364 |
| **DASH scores** |  |  |  |  |  |  |
| Unadjusted model | 0.66 | 0.51-0.85 | 0.002 | 0.85 | 0.79-0.91 | <.001 |
| Model 1^a^ | 0.77 | 0.60-0.99 | 0.044 | 0.89 | 0.82-0.96 | 0.002 |
| Model 2^b^ | 0.85 | 0.66-1.10 | 0.214 | 0.92 | 0.85-1.00 | 0.041 |
| Model 3^c^ | 0.82 | 0.61-1.09 | 0.163 | 0.90 | 0.83-0.98 | 0.022 |
| Model 4^d^ | 0.84 | 0.62-1.13 | 0.237 | 0.91 | 0.83-0.99 | 0.037 |

Abbreviations: CI, confidence interval; OR, Odds Ratio; MedD, mediterranean diet; DASH, dietary approaches to stop hypertension.

a Adjusted for gender, age, race/ethnicity, cohabitation, education, income-poverty ratio, body mass index and total energy.

b Adjusted for gender, age, race/ethnicity, cohabitation, education, income-poverty ratio, body mass index, total energy, smoking and pack-years.

c Adjusted for gender, age, race/ethnicity, cohabitation, education, income-poverty ratio, body mass index, total energy, smoking, pack-years, secondhand smoke and asthma.

d Adjusted for gender, age, race/ethnicity, cohabitation, education, income-poverty ratio, body mass index, total energy, smoking, pack-years, secondhand smoke, asthma and occupational exposure.

**eTable 4.** Associations of adherence to DASH and Mediterranean diet with COPD after excluding participants in cardiovascular disease, cancer or diabetes (n=16532).

|  |  | | | **Adjusted model** | | | | | | | | |
| --- | --- | --- | --- | --- | --- | --- | --- | --- | --- | --- | --- | --- |
| **Characteristic** | **Unadjusted model** | | | **Model 1 ^a^** | | | **Model 2 ^b^** | | | **Model 3 ^c^** | | |
|  | **OR** | **CI (95%)** | ***P*** | **OR** | **CI (95%)** | ***P*** | **OR** | **CI (95%)** | ***P*** | **OR** | **CI (95%)** | ***P*** |
| MedD scores |  |  |  |  |  |  |  |  |  |  |  |  |
| T1 | Ref |  |  | Ref |  |  | Ref |  |  | Ref |  |  |
| T2 | 0.81 | 0.61-1.06 | 0.121 | 0.75 | 0.56-0.99 | 0.042 | 0.82 | 0.62-1.09 | 0.167 | 0.92 | 0.69-1.24 | 0.592 |
| T3 | 0.78 | 0.62-0.99 | 0.039 | 0.79 | 0.62-1.00 | 0.047 | 0.97 | 0.75-1.25 | 0.826 | 1.05 | 0.79-1.38 | 0.749 |
| DASH scores |  |  |  |  |  |  |  |  |  |  |  |  |
| T1 | Ref |  |  | Ref |  |  | Ref |  |  | Ref |  |  |
| T2 | 0.70 | 0.55-0.89 | 0.004 | 0.71 | 0.56-0.90 | 0.005 | 0.74 | 0.58-0.95 | 0.018 | 0.74 | 0.58-0.94 | 0.014 |
| T3 | 0.56 | 0.44-0.73 | <.001 | 0.63 | 0.49-0.82 | 0.001 | 0.69 | 0.53-0.89 | 0.005 | 0.70 | 0.54-0.90 | 0.007 |

Abbreviations: CI, confidence interval; OR, Odds Ratio; MedD, mediterranean diet; DASH, dietary approaches to stop hypertension.

^a^ Adjusted for gender, age, race/ethnicity, cohabitation, education, income-poverty ratio, body mass index and total energy.

^b^ Adjusted for gender, age, race/ethnicity, cohabitation, education, income-poverty ratio, body mass index, total energy, smoking and pack-years.

^c^ Adjusted for gender, age, race/ethnicity, cohabitation, education, income-poverty ratio, body mass index, total energy, smoking, pack-years, secondhand smoke and asthma.

**eTable 5.** Associations of adherence to DASH and Mediterranean diet with FEV1 and FVC.

|  | **FEV1** | | | **FVC** | | |
| --- | --- | --- | --- | --- | --- | --- |
| **Characteristic** | **β** | **CI (95%)** | **P** | **β** | **CI (95%)** | **P** |
| **MedD scores** |  |  |  |  |  |  |
| Unadjusted model | -37.82 | -110.95 to 35.31 | 0.303 | -104.72 | -198.19 to -11.26 | 0.029 |
| Model 1^a^ | 50.33 | -5.39 to 106.06 | 0.075 | 4.49 | -51.66 to 60.64 | 0.872 |
| Model 2^b^ | 14.33 | -38.62 to 67.28 | 0.584 | 0.52 | -52.90 to 53.94 | 0.984 |
| Model 3^c^ | 8.53 | -43.12 to 60.19 | 0.737 | -3.13 | -57.05 to 50.79 | 0.906 |
| **DASH scores** |  |  |  |  |  |  |
| Unadjusted model | 166.95 | 100.01 to 233.89 | <.001 | 251.43 | 168.49 to 334.38 | <.001 |
| Model 1^a^ | 34.46 | -11.23 to 80.14 | 0.134 | 64.68 | 8.84 to 120.52 | 0.025 |
| Model 2^b^ | 18.48 | -28.12 to 65.09 | 0.424 | 60.96 | 4.81 to 117.11 | 0.034 |
| Model 3^c^ | 16.81 | -28.24 to 61.86 | 0.451 | 60.39 | 4.84 to 115.94 | 0.034 |

Abbreviations: OR, Odds Ratio; MedD, mediterranean diet; DASH, dietary approaches to stop hypertension.

a Adjusted for gender, age, race/ethnicity, cohabitation, education, income-poverty ratio, body mass index and total energy.

b Adjusted for gender, age, race/ethnicity, cohabitation, education, income-poverty ratio, body mass index, total energy, smoking and pack-years.

c Adjusted for gender, age, race/ethnicity, cohabitation, education, income-poverty ratio, body mass index, total energy, smoking, pack-years, secondhand smoke and asthma.

**eTable 6.** Associations of adherence to DASH and Mediterranean diet with FEV1 and FVC among participants without COPD and participants with COPD.

|  | **Participants without COPD** | | | **Participants with COPD** | | |
| --- | --- | --- | --- | --- | --- | --- |
| **Characteristic** | **β** | **CI (95%)** | **P** | **β** | **CI (95%)** | **P** |
| **MedD scores** |  |  |  |  |  |  |
| FVC | -15.10 | -65.89 to 35.68 | 0.547 | 140.15 | -99.92 to 380.23 | 0.241 |
| FEV1 | -6.05 | -52.86 to 40.76 | 0.793 | 167.78 | -6.41 to 399.66 | 0.149 |
| **DASH scores** |  |  |  |  |  |  |
| FVC | 60.47 | 7.70 to 120.17 | 0.047 | 16.81 | -151.27 to 184.88 | 0.839 |
| FEV1 | 16.62 | -30.52 to 63.76 | 0.476 | 5.74 | -128.01 to 139.48 | 0.930 |

Abbreviations: OR, Odds Ratio; MedD, mediterranean diet; DASH, dietary approaches to stop hypertension; FEV1, Forced expiratory volume in the first second; FVC, Forced vital capacity.

All β coefficient in this table are adjusted for gender, age, race/ethnicity, cohabitation, education, income-poverty ratio, body mass index, total energy, smoking, pack-years, secondhand smoke and asthma.

**eTable 7.** Characteristics of participants with and without complete data**.**

|  | **Participants with complete data (unweighted n=28095; weighted n=114364473)** | | **Participants with missing data (unweighted n=8157; weighted n=20079099)** | | ***P* ^a^** |
| --- | --- | --- | --- | --- | --- |
| **Characteristic** | **N** | **W%** | **N** | **W%** |  |
| Gender |  |  |  |  | 0.031 |
| Male | 13845 | 47.5 | 3827 | 45.6 |  |
| Female | 14250 | 52.5 | 4330 | 54.4 |  |
| Age |  |  |  |  | <.001 |
| 40-50 | 7380 | 32.1 | 1684 | 28.4 |  |
| 50-60 | 6484 | 28.9 | 1617 | 26.4 |  |
| >60 | 14231 | 39.0 | 4856 | 45.2 |  |
| Race/ethnicity |  |  |  |  | <.001 |
| Non-Hispanic White | 13608 | 74.6 | 3278 | 62.6 |  |
| Non-Hispanic Black | 5818 | 9.8 | 1779 | 13.7 |  |
| Mexican American | 4382 | 5.6 | 1379 | 7.6 |  |
| Other Hispanic | 2153 | 4.3 | 768 | 6.1 |  |
| Other Race | 2134 | 5.7 | 953 | 9.9 |  |
| Cohabitation |  |  |  |  | <.001 |
| No | 10444 | 31.6 | 3425 | 37.1 |  |
| Yes | 17651 | 68.4 | 4391 | 62.9 |  |
| Missing | NA |  | 341 |  |  |
| Education |  |  |  |  | <.001 |
| High school | 6524 | 24.4 | 1807 | 24.0 |  |
| Less than high school | 7933 | 17.0 | 3067 | 26.0 |  |
| More than high school | 13638 | 58.6 | 3278 | 50.0 |  |
| Missing | NA |  | 5 |  |  |
| Income-poverty ratio |  |  |  |  | <.001 |
| ≤ 2 | 12523 | 29.9 | 2375 | 43.8 |  |
| ＞2 | 15572 | 70.1 | 2040 | 56.2 |  |
| Missing | NA |  | 3742 |  |  |
| BMI |  |  |  |  | <.001 |
| 18.5-25 | 6846 | 25.2 | 1498 | 28.4 |  |
| <18.5 | 336 | 1.2 | 94 | 1.8 |  |
| 25-30 | 10050 | 35.4 | 1928 | 35.1 |  |
| >30 | 10863 | 38.3 | 1941 | 34.8 |  |
| Missing | NA |  | 2696 |  |  |
| Total energy, kcal/day |  |  |  |  | <.001 |
| Mean ± SE | 25607 | 2042.17 ± 7.32 | 3903 | 1931.92 ± 18.88 |  |
| Missing | NA |  | 4254 |  |  |
| Smoking status |  |  |  |  | <.001 |
| No | 14058 | 50.6 | 4271 | 51.1 |  |
| Yes | 14037 | 49.4 | 3834 | 48.9 |  |
| Missing | NA |  | 55 |  |  |
| Secondhand Smoke |  |  |  |  | <.001 |
| No | 22318 | 80.1 | 5988 | 78.4 |  |
| Yes | 5777 | 19.9 | 1532 | 21.6 |  |
| Missing | NA |  | 637 |  |  |
| Asthma |  |  |  |  | <.001 |
| No | 24577 | 87.0 | 7161 | 87.5 |  |
| Yes | 3518 | 13.0 | 957 | 12.5 |  |
| Missing | NA |  | 39 |  |  |

Abbreviations: CI, confidence interval; BMI, body mass index; MedD, mediterranean diet; DASH, dietary approaches to stop hypertension.

^a^ P values are design-adjusted Rao-Scott Pearson χ2 test for categorical variables and T test for continuity variables.

**eTable 8.** DASH diet composition and scoring criteria.

| **Nutrient** | **Score Target (1 point)** | **Intermediate Target (0.5 point)** | **Average score in our study** |
| --- | --- | --- | --- |
| saturated fat | 6% of energy | 11% of energy | 0.276 |
| total fat | 27% of energy | 32% of energy | 0.263 |
| protein | 18% of energy | 16.5% of energy | 0.332 |
| cholesterol | 71.4 mg/1000 kcal | 107.1 mg/1000 kcal | 0.335 |
| fiber | 14.8 mg/1000 kcal | 9.5 mg/1000 kcal | 0.202 |
| magnesium | 238 mg/1000 kcal | 158 mg/1000 kcal | 0.227 |
| calcium | 590 mg/1000 kcal | 402 mg/1000 kcal | 0.365 |
| potassium | 2238 mg/1000 kcal | 1534 mg/1000 kcal | 0.197 |
| sodium | 1143 mg/1000 kcal | 1286 mg/1000 kcal | 0.291 |

**eTable 9.** Mediterranean diet composition and scoring criteria.

| **Food category** | **Score Target (2 point)** | **Intermediate Target (1 point)** | **Average score in our study** |
| --- | --- | --- | --- |
| fruits | 2 CE | 1 CE | 0.449 |
| vegetables | 1 CE | 0.5 CE | 0.833 |
| legumes | 140 g | 70 g | 0.124 |
| cereals | 195 g | 130 g | 0.018 |
| fish | 250 g | 100 g | 0.063 |
| dairy | 270 g | 180 g | 1.236 |
| olive oil | 28 g | 14 g | 0.005 |
| meat | 80 g | 120 g | 1.420 |
| alcohol | 12 g | 24 g | 1.645 |
